# Model Development for Threshold Voltage Stability Dependent on High Temperature Operations in Wide-Bandgap GaN-Based HEMT Power Devices

**DOI:** 10.3390/mi9120658

**Published:** 2018-12-14

**Authors:** Huolin Huang, Feiyu Li, Zhonghao Sun, Yaqing Cao

**Affiliations:** 1 School of Optoelectronic Engineering and Instrumentation Science, Dalian University of Technology, Dalian 116024, China; lifeiyu@mail.dlut.edu.cn (F.L.); sunzhonghao@mail.dlut.edu.cn (Z.S.); cyqtmxk@mail.dlut.edu.cn (Y.C.); 2 Key Laboratory for Micro/Nano Technology and System of Liaoning Province, Dalian University of Technology, Dalian 116024, China

**Keywords:** threshold voltage (*V*_th_) stability, gallium nitride (GaN), high electron mobility transistors (HEMTs), analytical model, high-temperature operation

## Abstract

Temperature-dependent threshold voltage (*V*_th_) stability is a significant issue in the practical application of semi-conductor power devices, especially when they are undergoing a repeated high-temperature operation condition. The *V*_th_ analytical model and its stability are dependent on high-temperature operations in wide-bandgap gallium nitride (GaN)-based high electron mobility transistor (HEMT) devices that were investigated in this work. The temperature effects on the physical parameters—such as barrier height, conduction band, and polarization charge—were analysed to understand the mechanism of *V*_th_ stability. The *V*_th_ analytical model under high-temperature operation was then proposed and developed to study the measurement temperatures and repeated rounds dependent on *V*_th_ stability. The validity of the model was verified by comparing the theoretical calculation data with the experimental measurement and technology computer-aided design (TCAD) simulation results. This work provides an effective theoretical reference on the *V*_th_ stability of power devices in practical, high-temperature applications.

## 1. Introduction

Gallium nitride (GaN)-based high electron mobility transistors (HEMTs) have demonstrated a great potential in the fields of power electronics, mainly owing to their large semi-conductor bandgap (~3.4 eV), low intrinsic carrier concentration, and high-density two dimensional electron gas (2DEG) (>10^13^ cm^−2^), along with their high electron mobility (>2000 cm^2^∙V∙s^−1^) at the AlGaN/GaN heterojunction interface [1,2,3]. In comparison with Si or GaAs-based field-effect transistors (FETs), the wide-bandgap GaN-based devices have lower specific on-resistance and a faster ON/OFF switching speed. Therefore, they are well suited for high power switching applications in renewable energy systems, smart power grids, industrial motors, and the like. In particular, in the last decade, GaN-based HEMTs have attracted significant interest for high-frequency applications because the devices exhibit good noise properties comparable to GaAs-based HEMTs, but with the advantage of having a much higher input power robustness [4,5,6,7]. GaN-based HEMTs with a selected short gate length have overcome laterally diffused metal oxide semiconductor (LDMOS)-based transistors for the applications above the L-band owing to their higher frequency capabilities. Furthermore, GaN-based devices are also very promising when employed in various high-temperature environments, such as aerospace turbines and automotive internal combustion engines [8,9,10]. As a result of the considerable and worldwide attention given to the GaN techniques, they have achieved rapid and remarkable progress.

However, there are still several issues that need to be worked out for the practical application of GaN-based products [11,12]. An urgent task is to investigate the temperature-dependent performance stability, for instance, the device threshold voltage (*V*_th_) stability [13]. To date, there has been much research on the temperature-dependent analytical model, focusing on investigating a device’s temperature-dependent output current–voltage characteristics and equivalent circuits. The temperature-dependent semi-conductor interface state and electron mobility have been extensively investigated [14,15,16,17]. The equivalent circuit modelling of GaN-based HEMTs as a function of ambient temperature has been accurately established, which is meaningful since the equivalent circuit is a very useful tool for circuit designers [18,19]. Besides, some work has studied the *V*_th_ variation according to the measurement temperature [20,21,22]. However, there is still a lack of sufficient research on the *V*_th_ analytical model and, in particular, on its stability when subjected to repeated high temperature operations, which might result in an unrecoverable impact on the material properties, for example the interface trap density. 

This paper systematically investigates the *V*_th_ analytical model and the stability mechanism when subjected to the different measurement temperatures and repeated rounds in GaN-based HEMTs. Considering that a relatively large gate length of 2 µm was employed in the fabricated devices, the direct current (DC) characteristics are analysed in this work. The physical model was developed step-by-step by first building and analysing the *V*_th_ model in the conventional Schottky gate, with an emphasis on the influence of the physical parameters of the basic device on the *V*_th_. The model was then analysed in the metal–insulator–semiconductor (MIS) gate structure, with an emphasis on the influence of the charged interface traps. A series of *V*_th_ values for the fabricated HEMTs were derived after repeating high-temperature measurements from room temperature to high-temperature for several rounds. The effects of the high temperature on the physical parameters, such as barrier height, conduction band, Fermi level, polarization charge, and interface traps were analysed to understand the *V*_th_ stability mechanism. Both the experimental measurement of the device and technology computer-aided design (TCAD) simulation work were carried out and the validity of the model was finally verified.

## 2. Physical Mechanism and Threshold Voltage (V_th_) Analytical Model

Schottky contact is a basic element in power electronic devices. Therefore, physical modelling work starts with an investigation of the characteristics of simple Schottky-gate HEMT devices. The detailed study of effects of high-temperature on the Schottky-gate devices was carried out to understand the role of the basic physical parameters, such as barrier height, conduction band, Fermi level and polarization charges in the operation at high-temperature. Then, more complicated structures with the MIS gate were involved to analyse the effects of the interface traps beneath the gate, considering that the MIS gate structure has a much higher density of interface traps between the dielectric and AlGaN barrier layer than the Schottky gate. These charged traps could induce an instability of the *V*_th_ value, especially when the devices were operated under the high-temperature condition [23,24,25]. The physical mechanisms of the trap density variation with the different operation temperatures and sequences were discussed and the analytical model was proposed and developed.

### 2.1. Schottky-Gate AlGaN/GaN High Electron Mobility Transistors (HEMTs)

By taking into account the combined effects of the device’s physical parameters at different temperatures, such as Schottky barrier, polarization charges, energy band and doping-induced charges, the equation to generally describe the *V*_th_ of the Schottky-gate devices can be modified and expressed as below [26].

(1) $$\begin{array}{cc} V_{\mathrm{th}1}\left(T\right)=\frac{\varphi_{b}\left(T\right)}{q} & -\frac{\Delta E_{C}\left(T\right)}{q}+\frac{E_{F0}\left(T\right)}{q}\\ & -\frac{qd\sigma_{pol}\left(T\right)}{\varepsilon_{\mathrm{AlGaN}}}\\ & -\frac{qN_{d}\left(T\right)d^{2}}{2\varepsilon_{\mathrm{AlGaN}}} \end{array}$$

In the equation, $\varphi_{b}$ is the contact barrier height between the gate metal and AlGaN barrier layer. $\Delta E_{C}$ is the conduction band offset at the AlGaN/GaN interface. $E_{F0}$ is the energy difference between the intrinsic Fermi level and the conduction band edge of GaN bulk. $\sigma_{pol}$ is the net polarization charge at the AlGaN/GaN interface. *N_d_* is the doping concentration in the AlGaN layer, and $\varepsilon_{\mathrm{AlGaN}}$ is the AlGaN permittivity. For simplicity, the AlGaN doping-induced effect on *V*_th_ can be ignored in the unintentionally doped AlGaN/GaN structure, due to its low intrinsic carrier concentration in the wide-bandgap semiconductor. Therefore, only the effects of the front four terms in Equation (1) were discussed here. 

#### 2.1.1. Schottky Barrier Height

The Schottky barrier height under zero bias can be determined by the expression [27]:

(2) $$\varphi_{b}\left(T\right)=\eta \varphi_{\mathrm{EB}}\left(T\right)-\left(\eta -1\right)\left[\Delta E_{C}-E_{F0}\left(T\right)\right]$$

where $\varphi_{\mathrm{EB}}$ and η are the effective Schottky barrier height and ideality factor related to the applied electric field, respectively. They can be deduced from the experimental data using the expression $\ln\left(I_{GS}/aA^{*}T^{2}\right)=q\left(V_{GS}-\eta \varphi_{\mathrm{EB}}\right)/\eta kT$. Here, *I_GS_* and *V_GS_* are the gate-to-source current and voltage, *a* is the gate contact area, and A^*^ (= 28.4 A∙cm^−2^∙K^−2^) is the effective Richardson constant.

#### 2.1.2. Conduction Band Offset

Conduction band offset ($\Delta E_{C}$) plays an important role in determining the 2DEG density at the AlGaN/GaN interface. $\Delta E_{C}$ at different temperatures can be expressed as below [28,29].

(3) $$\Delta E_{C}\left(T\right)=0.7\left[E_{g}^{\mathrm{AlGaN}}\left(T\right)-E_{g}^{\mathrm{GaN}}\left(T\right)\right]$$

where the material bandgap with the different Al composition *x* can be obtained by

(4) $$E_{g}^{\mathrm{AlGaN}}\left(T,x\right)=xE_{g}^{\mathrm{AlN}}\left(T\right)+\left(1-x\right)E_{g}^{\mathrm{GaN}}\left(T\right)-x\left(1-x\right)$$

(5) $$E_{g}^{\mathrm{AlN}}\left(T\right)=6.31-1.80\times 10^{-3}\times \frac{T^{2}}{T+1462}$$

(6) $$E_{g}^{\mathrm{GaN}}\left(T\right)=3.51-9.09\times 10^{-4}\times \frac{T^{2}}{T+830}$$

#### 2.1.3. Fermi Energy

The Fermi energy level in GaN semiconductor can be defined by the empirical equation, as below [26].

(7) $$E_{F0}\left(T\right)=k_{1}\left(T\right)+k_{2}\left(T\right)n_{s}^{1/2}\left(T\right)+k_{3}\left(T\right)n_{s}$$

where *k*_1_, *k*_2_, and *k*_3_ are the temperature-dependent parameters and *n_s_* is the sheet charge density.

#### 2.1.4. Polarization Charge

High-density 2DEG will be formed at the AlGaN/GaN interface due to the existence of the large spontaneous and piezoelectric polarization induced electric field that can be determined by the elastic and piezoelectric constants of the materials in the c-axis direction. The sheet charge density induced by the net polarization will be deduced directly by the polarization expression from the published material parameters [28]. Although the net polarization-induced charge density is obviously changed depending on the Al composition and AlGaN barrier thickness, the pyroelectric coefficients that describe the changes in polarization depending on the temperature were found to be very small [30,31]. Chang et al. proved that the effect of pyroelectric coefficients on the channel current at high temperatures is negligible [3]. Therefore, the net polarization charge density was considered to be constant at a temperature less than 150 °C in this work.

### 2.2. Metal-Insulator-Semiconductor (MIS)-Gate AlGaN/GaN HEMTs

The *V*_th_ shift occurs more often in MIS-gate HEMTs than in Schottky-gate devices. The dominant reason is the existence of high-density traps at the dielectric/AlGaN interface near the gate. Although it is generally accepted that the *V*_th_ instability is induced by the combined effects of several trap species that are located in the GaN bulk or dielectric oxide or at the dielectric/AlGaN interface, the influence of the dielectric/AlGaN interface traps should be the most significant, given that it has the highest trap density (10^12^–10^13^ cm^−2^) and shortest distance to the 2DEG channel.

The band energy level of these traps plays an important role in determining the *V*_th_ values of the devices when they are operated at different high temperatures. Moreover, the amount of variation of these traps also affects the consistency of the *V*_th_ during repeated high-temperature operation. The trap amount will finally decrease and become stable after high-temperature measurements for several rounds. The repeated carrier injection and hopping among the traps due to thermal diffusion or the tunnelling process with the help of an electric field, are considered to be the main physical mechanisms that finally stabilize the trap energy level and amount after repeated high-temperature current–voltage *(I-V)* measurements. Therefore, the effects of the trap energy level and amount at the dielectric/AlGaN interface are included in this section to redefine the *V*_th_ in the MIS-gate AlGaN/GaN HEMTs. The *V*_th_ can be derived from the expressions below.

(8) $$\begin{array}{cc} V_{\mathrm{th}2}\left(T\right)=\frac{\varphi_{b}\left(T\right)}{q} & -\frac{\Delta E_{C1}\left(T\right)}{q}+\frac{E_{F0}\left(T\right)}{q}-\frac{\Delta E_{C2}\left(T\right)}{q}-\frac{qd\sigma_{pol}\left(T\right)}{\varepsilon_{\mathrm{AlGaN}}}-\frac{qd_{OX}\sigma_{pol}\left(T\right)}{\varepsilon_{OX}}\\ & -\frac{qd_{OX}N_{IT}\left(T\right)}{\varepsilon_{OX}} \end{array}$$

(9) $$N_{IT}(T,t)=N_{IT0}(T)\left[1-k_{t}\exp(-\frac{1}{t})\right]$$

(10) $$N_{IT0}(T)=N_{IT0}\left(T=25\;{}^{\circ}C)\exp[-\frac{\Delta E_{IT}\left(T\right)}{kT}\right]$$

(11) $$\tau_{n}=\frac{1}{NCv\sigma_{n}}\exp\left(\frac{E_{C}-E_{D}}{kT}\right)$$

Here $d_{OX}$ and $\varepsilon_{OX}$ are the thickness and dielectric constant of the gate oxide, respectively. $\Delta E_{C1}$ and $\Delta E_{C2}$ are the conduction band offsets at the AlGaN/GaN and dielectric/AlGaN interfaces, respectively. $N_{IT}\left(T\right)$ is the charged trap density at the dielectric/AlGaN interface which is varied with the measurement temperature and operation count at high temperature up to 150 °C. $N_{IT0}(T)$ is the initial interface trap density. *k_t_* is the coefficient to describe the effect of the high-temperature measurement count on the trap amount and *t* is the repeated measurement rounds. $\Delta E_{IT}\left(T\right)$ is the variation value of the trap energy level at different measurement temperatures. *N*_C_, *v*, *σ*_n_, and *E*_D_ are the effective density of states in the conduction band, the thermal velocity of electrons, the capture cross section and the energetic location of the traps, respectively. *E*_C_ − *E*_D_ = 0.37 eV and *v* = 6 × 10^6^ cm∙s^−1^ at AlGaN surface were used in the work [12].

The front six terms in Equation (8) describe the combined effects that originated from the basic physical parameters, i.e., the Schottky barrier, energy band, and polarization-induced charges on the *V*_th_ variation in the MIS-gate HEMTs, which are similar and consistent with the Schottky-gate case discussed earlier. The term $qd_{OX}\sigma_{pol}\left(T\right)/\varepsilon_{OX}$ in Equation (8) indicates an additional effect of the polarization-induced charges on the *V*_th_ by adding the gate oxide dielectric in the HEMTs. As mentioned, the effect was negligible since the variation of the polarization charge density with the temperature was small. The last term in Equation (8) related to the interface traps will play a key role in how the *V*_th_ changes with the measurement temperature and repeated rounds. Equation (9) is proposed to govern the amount variation of the charged interface traps, with increasing high temperature measurement rounds. Initially (*t*~0), *N*_IT_ is equal to $N_{IT0}\left(T\right)$, corresponding to the case without any high-temperature operation. Then *N*_IT_ decreased with the increasing measurement count *t* and finally the trap amount will be constant, and the *V*_th_ value will be stable from Equation (9). The coefficient *k_t_* is used to distinguish the effects of different measurement temperatures on the trap amount during the repeated high-temperature measurements. In Equation (10), the trap energy level is dependent on the temperature. The effective trap amount can be determined by the variation of the trap energy level with the increasing measurement temperature. In this work, the Shockley-Read-Hall (SRH) model was employed to describe the physical charge-trapping behaviour through the deep defect level in the gap. Several mathematical models were used to determine the temperature-dependent parameters, such as the material bandgap, Fermi level, polarization charge, carrier mobility and carrier lifetimes (or time constant) in the simulation work.

## 3. Experiments and Results

### 3.1. Fabrication and Measurement Process

**Figure 1:**
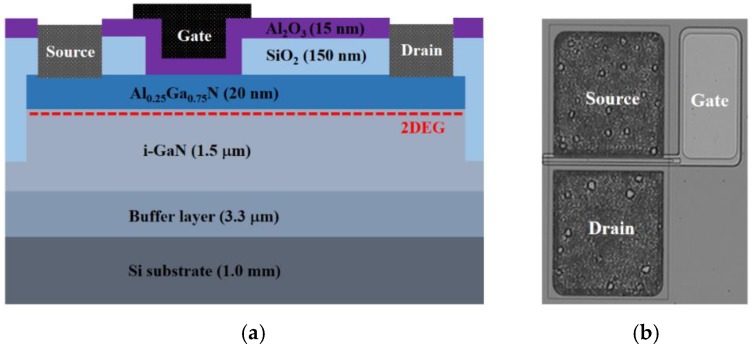
(**a**) Cross-sectional schematic and (**b**) optical microscopy image of the fabricated AlGaN/GaN metal–insulator–semiconductor (MIS)-gate high electron mobility transistors (HEMTs).

The schematic and optical microscopy image of the typical MIS-gate HEMTs are shown in Figure 1a,b, respectively. The specifics of the device structure are contained in Figure 1a. The fabrication work of the devices began with the mesa isolation by selectively etching the epitaxial layers with 300 nm depth. Ideal source and drain Ohmic contacts were achieved by depositing Ti/Al/Ni/Au (25 nm/125 nm/45 nm/55 nm) metal alloys using the E-beam system and annealing at 850 °C for 30 s in N_2_. SiO_2_ film with 150 nm thickness was deposited using a plasma-enhanced chemical vapour deposition (PECVD) system for surface passivation. Then the gate window with 2 µm length and 200 µm width was defined by photolithography, followed by the removal of the SiO_2_ film by wet etching. The Al_2_O_3_ gate dielectric layer with 15 nm thickness was deposited by the atomic layer deposition (ALD) system. Ni/Au metals were then deposited using the E-beam system for the gate electrode.

The *I*-*V* characteristics of the fabricated devices were measured at gradually increasing temperatures from 25 °C to 150 °C with a step of 25 °C using an Agilent B1505A semi-conductor device analyser (Agilent, Santa Clara, CA, USA). The measurement process was repeated for several rounds, and the testing platform was always cooled down naturally to room temperature before starting a new measurement round. Each measurement point was maintained at a setting temperature for 10 min in ambient air and then the drain current-gate voltage (*I*_d_*-V*_g_) transfer curves were measured. The average measurement time for each *I*-*V* curve was around 2 s. The device-related physical parameters employed in the modelling and TCAD simulation processes were calibrated by benchmarking the device *I*-*V* characteristics with the measurement data. The typical parameter values are listed in Table 1 [12,32]. Verification was made by the simulation and laboratory measurement data to support the validity of the proposed model in the paper.

### 3.2. Model Verification and Discussion

#### 3.2.1. Results for Schottky-Gate AlGaN/GaN HEMTs

**Figure 2:**
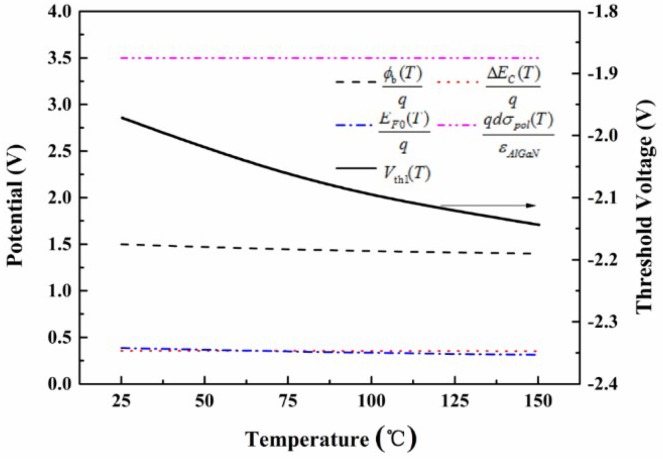
Specific contributions of the main physical parameters to the threshold voltage (*V*_th_) variations in the Schottky-gate AlGaN/GaN HEMTs using the analytical model (dash lines). The solid line shows the general *V*_th_ value that varied with the temperature.

Figure 2 shows the detailed effects of the main physical parameters that varied with the temperature on the *V*_th_ stability. The *V*_th_ shifted slightly towards the negative direction as the temperature increased. The general effect on the *V*_th_ variation in the Schottky-gate HEMTs is displayed in Figure 2. The variation value of *V*_th_ was found to be around 0.15 V in the Schottky-gate devices when the temperature changed from 25 °C to 150 °C, which demonstrates that the *V*_th_ shift in Schottky-gate devices is relatively small.

#### 3.2.2. Results for the MIS-Gate AlGaN/GaN HEMTs

**Figure 3:**
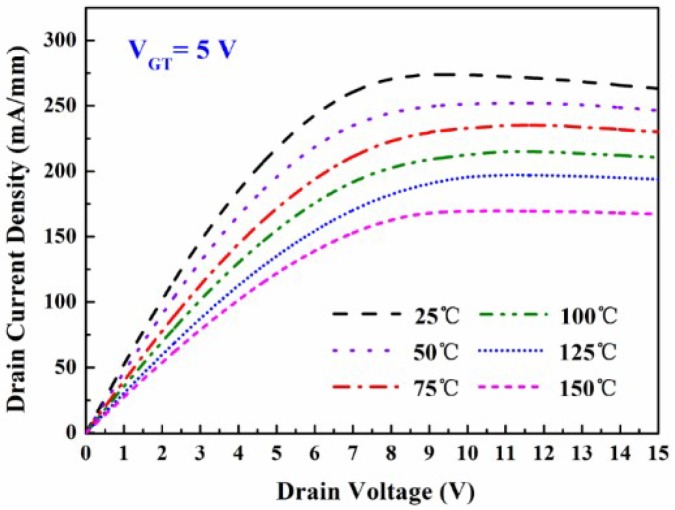
Measurement of the temperature-dependent direct current (DC) output characteristics of the fabricated MIS-gate HEMTs. *V*_GT_ = *V*_g_ – *V*_th_ = 5 V was employed in the measurements.

**Figure 4:**
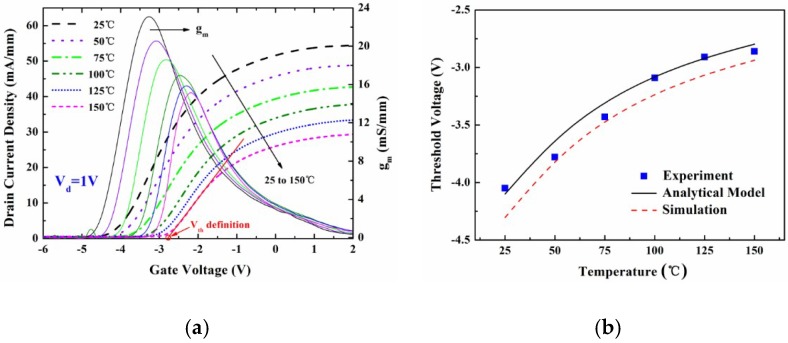
(**a**) Temperature-dependence of the typical device transfer characteristics and gate transconductance *g*_m_ in the MIS-gate HEMTs, and (**b**) the *V*_th_ data changed with the increasing temperatures in the experimental measurement, analytical calculation using the model, and technology computer-aided design (TCAD) simulation work.

Figure 3 shows the measurement of the temperature-dependent DC output characteristics of the fabricated MIS-gate HEMTs. The drain current density decreased with the increasing temperature. This was mainly attributed to the degeneration of the electron mobility in the 2DEG channel induced by the thermal lattice vibration scattering. The source-to-drain channel conductance *g*_ds_ was found to be 52.3 mS/mm, 46.6 mS/mm, 39.8 mS/mm, 35.6 mS/mm, 30.4 mS/mm, and 27.5 mS/mm, respectively. Figure 4a shows the typical *I*_d_-*V*_g_ curves and gate transconductance *g*_m_ characteristics of the fabricated MIS-gate, AlGaN/GaN HEMTs. The *g*_m_ peak declined by 34.7% when the measurement temperature was up to 150 °C, which suggests a reduction of the device switching frequency. 

The *V*_th_ values were determined by extrapolating the linear portion of the plot of the drain current density (*I*_d_) to the *x* axis (*V*_g_). The intercept at the voltage axis was defined as the *V*_th_ in this paper. *V*_d_ = 1 V was used in the measurement process for the device transfer characteristics. An illustration of *V*_th_ definition is provided in Figure 4a. Figure 4b displays a trend of the *V*_th_ data variation with various temperatures. It was found that the measured *V*_th_ data shifted from −4.1 V to −2.7 V, towards more positive values with the increasing temperature. The big variance likely mainly originated from the combined effects of the change of interface trap number and the shift of the trap energy level during the process of the temperature increasing. More electrons may be captured by high-density ionized donor-like traps at the Al_2_O_3_/AlGaN interface beneath the gate at high temperatures. Thus, the number of the interface positive charges decreased, which resulted in the shift of the *V*_th_. Equation (10) was employed to clearly describe the relationship between the trap density and the operation temperature. Both the calculation results based on the proposed analytical model and the simulation work agree well with the experimental data, which supports the validity of the physical model.

**Figure 5:**
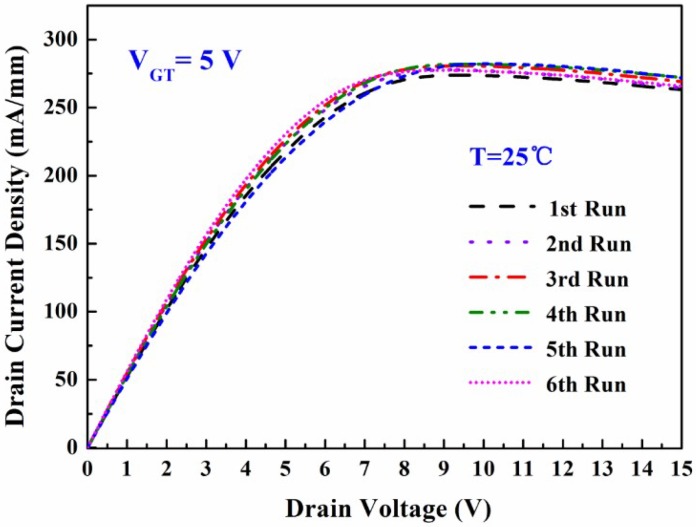
Repeated measurement-dependence of the DC output characteristics in the fabricated MIS-gate HEMTs.

**Figure 6:**
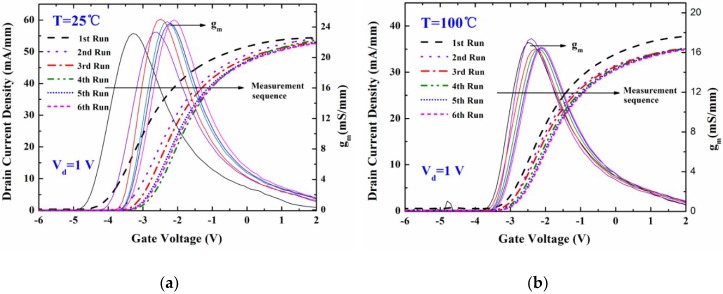
Repeated measurement dependence of the typical device transfer characteristics and gate transconductance *g*_m_ in the MIS-gate HEMTs at (**a**) 25 °C and (**b**) 100 °C, respectively.

**Figure 7:**
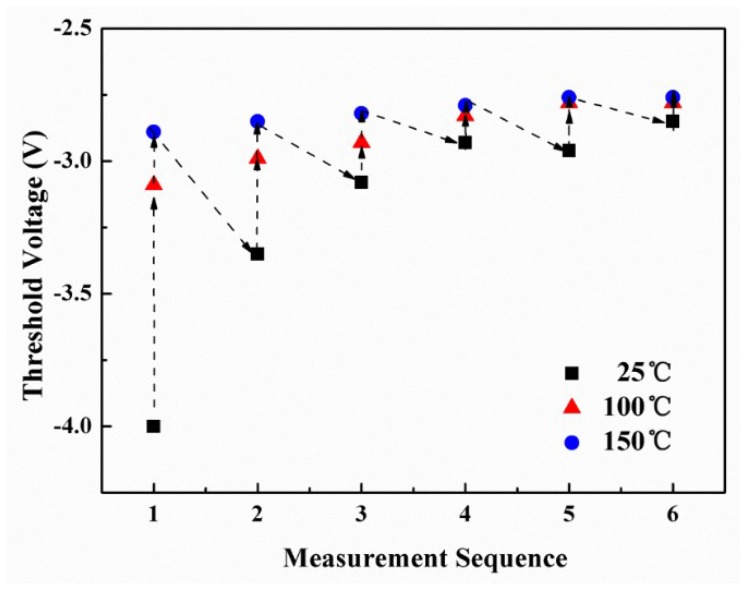
The *V*_th_ data changed with the repeated measurement rounds in the MIS-gate HEMTs. The data were measured at gradually increasing temperatures from 25 °C to 150 °C and the measurement process was repeated for several rounds. The dotted lines and arrows show the measurement sequence.

Figure 5 shows the repeated measurement-dependence of the DC output characteristics of the fabricated MIS-gate HEMTs, which demonstrates that the drain current remained almost unchanged even after several rounds of *I*-*V* measurements. Figure 6 shows the typical *I*_d_-*V*_g_ curves and *g*_m_ characteristics of the fabricated MIS-HEMTs, dependent on the repeated measurement rounds. The *g*_m_ peak changed slightly after several measurement rounds and the variation was less than 7.8%. Figure 7 displays the *V*_th_ values and the measurement flow. The data were measured at gradually increased temperatures from 25 °C to 150 °C and then the sample was cooled down naturally to room temperature before starting a new measurement round. The measurement process was repeated for several rounds until the *V*_th_ value was stable. Thus, the repeated measurement dependence of the *V*_th_ was achieved, as shown in Figure 7. A big variance around 1.1 V was found at the beginning, while the *V*_th_ value was kept nearly constant and only a small change of 0.1 V was found after several rounds of high-temperature measurements. This indicates that most ionized donor-like traps capture the free electrons and hence the number of charged traps is finally close to constant. 

**Figure 8:**
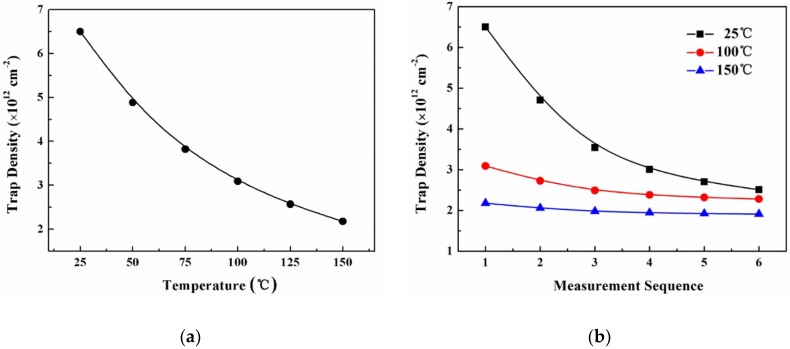
Trap density data at the Al_2_O_3_/AlGaN interface employed in the analytical model and TCAD simulation, dependent on (**a**) the operation temperature and (**b**) measurement sequence.

**Figure 9:**
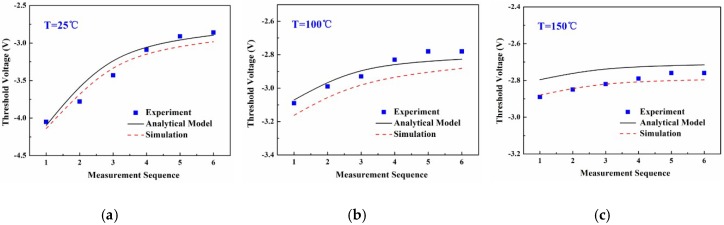
The *V*_th_ data changed with the measurement sequence from the experimental measurement, analytical calculation using the model, and TCAD simulation work when operated at (**a**) 25 °C, (**b**) 100 °C, and (**c**) 150 °C, respectively.

Figure 8 shows the employed trap density data at the Al_2_O_3_/AlGaN interface in the analytical model and TCAD simulation, which are dependent on the measurement temperature and sequence. The amount of variation of these charged traps greatly affected the consistency of the *V*_th_ during the repeated high-temperature operations. The charged trap amount was assumed to decrease with the increasing measurement rounds and finally became stable in the proposed physical model. The repeated carrier injection and hopping among the traps by the thermal diffusion and/or tunnelling process in the measurements, were considered to be the main physical mechanism responsible for the amount of variation of the charged traps. This process might repair some defects to some extent, and finally stabilizes the charged trap number after repeated high-temperature *I*-*V* measurements. Future work should explore whether the applied low electric field at high temperatures helps to repair the interface defects around the gate. Equation (9) in the manuscript was employed to govern the amount of variation of the traps with the increasing high-temperature measurement rounds. Figure 9 displays the *V*_th_ values that changed with the measurement sequence based on the experimental measurement, analytical model, and TCAD simulation work. The results were basically consistent and the average mismatch for the *V*_th_ value was kept within 5%, which again supports the validity of the physical model.

## 4. Conclusions

GaN-based HEMT devices were fabricated and measured to investigate the effects of the operation temperature and repeated rounds on the *V*_th_ stability. The *V*_th_ analytical model was proposed and developed to study the mechanism of the *V*_th_ variations in the repeated high-temperature operations. The combined effects of the amount of change of the interface charged traps and the shift of the trap energy level were considered to be the main reason for the *V*_th_ shift. The validity of the proposed analytical model was verified by experimental measurement and TCAD simulation results. The work can assist the engineers find a better understanding of the *V*_th_ stability of power devices in practical high-temperature applications.

## Figures and Tables

**Table 1:** Summary of the typical parameters adopted in this work. 2DEG–two dimensional electron gas.

Parameters	Descriptions	Values
*n*_s_	2DEG sheet density	6.7 × 10^12^ cm^−2^
$\sigma_{pol}$	Sheet density of polarization charges at the AlGaN/GaN interface	1.0 × 10^13^ cm^−2^
*N*_IT_	Donor-like trap density at the gate dielectric/AlGaN interface	6.5 × 10^12^ cm^−2^
*µ*_n_	Electron mobility	1050 cm^2^∙V∙s^−1^
*φ*_Ni_	Ni work function	5.1 eV
$\varepsilon_{OX}$	Al_2_O_3_ dielectric constant	9.0
$\varepsilon_{\mathrm{AlGaN}}$	AlGaN dielectric constant	10.3 (when x = 0.25)
$\varphi_{b}$	Barrier height	1.5 eV for Schottky and 3.7 eV for MIS gates
$E_{F0}$	Fermi level from GaN conduction band edge	0.39 eV
$\Delta E_{C2}$	Conduction band offset at the Al_2_O_3_/AlGaN interface	1.10 eV
$\Delta E_{C1}$	Conduction band offset at the AlGaN/GaN interface	0.36 eV
*k_t_*	Stability coefficient	0.75 at 25 °C; 0.32 at 100 °C; 0.15 at 150 °C
*τ*_n_	Time constant at 25 °C	8.6 × 10^−7^ s
*N*_C_	Conduction band state density	2.2 × 10^18^ cm^−3^ for GaN and 4.1 × 10^18^ cm^−3^ for AlN
*σ*_n_	Capture cross section of the traps	1.0 × 10^−13^ cm^2^ for both GaN and AlN
*α*	Temperature coefficient	9.09 × 10^−4^ eV∙K^−1^ for GaN and 1.80 × 10^−3^ eV∙K^−1^ for AlN
